# The Dose-Dependent Effect of Nesiritide on Renal Function in Patients with Acute Decompensated Heart Failure: A Systematic Review and Meta-Analysis of Randomized Controlled Trials

**DOI:** 10.1371/journal.pone.0131326

**Published:** 2015-06-24

**Authors:** Bo Xiong, Chunbin Wang, Yuanqing Yao, Yuwen Huang, Jie Tan, Yin Cao, Yanke Zou, Jing Huang

**Affiliations:** Department of Cardiology, The Second Affiliated Hospital of Chongqing Medical University, Chongqing, China; Cardiff University, UNITED KINGDOM

## Abstract

**Background:**

Conflicting renal effects of nesiritide have been reported in patients with acute decompensated heart failure. To answer this controversy, we performed a meta-analysis of randomized controlled trials to evaluate the influence of nesiritide on renal function in patients with acute decompensated heart failure.

**Methods:**

Articles were obtained from PubMed, Medline, Cochrane Library and reference review. Randomized controlled studies that investigated the effects of continuous infusion of nesiritide on renal function in adult patients with acute decompensated heart failure were included and analyzed. Fixed-effect model was used to estimate relative risk (RR) and weight mean difference (WMD). The quality assessment of each study, subgroup, sensitivity, and publication bias analyses were performed.

**Results:**

Fifteen randomized controlled trials were eligible for inclusion. Most of included studies had relatively high quality and no publication bias was found. Overall, compared to control therapies, nesiritide might increase the risk of worsening renal function in patients with acute decompensated heart failure (RR 1.08, 95% CI 1.01–1.15, *P* = 0.023). In subgroup analysis, high-dose nesiritide strongly associated with renal dysfunction (RR 1.54, 95% CI 1.19-2.00, *P* = 0.001), but no statistical differences were observed in standard-dose (RR 1.04, 95% CI 0.98-1.12, *P* = 0.213), low-dose groups (RR 1.01, 95% CI 0.74-1.37, *P* = 0.968) and same results were identified in the subgroup analysis of placebo controlled trials. Peak mean change of serum creatinine from baseline was no significant difference (WMD -2.54, 95% CI -5.76-0.67, *P* = 0.121).

**Conclusions:**

In our meta-analysis, nesiritide may have a dose-dependent effect on renal function in patients with acute decompensated heart failure. High-dose nesiritide is likely to increase the risk of worsening renal function, but standard-dose and low-dose nesiritide probably have no impact on renal function. These findings could be helpful to optimize the use of nesiritide in clinical practice.

## Introduction

Worsening renal function (WRF) is prevalent in patients with acute decompensated heart failure (ADHF), and it may independently predict a worse outcome as well as increase the mortality of ADHF patients [1–3]. Hence, preservation of renal function is one of the major therapeutic goals in the management of ADHF.

Nesiritide, a recombinant B-type natriuretic peptide (BNP) with a potent vasodilatory property, has been approved for the treatment of ADHF, on the basis of its ability to reduce pulmonary-capillary wedge pressure and improve dyspnea [4, 5]. After approval, numerous studies have investigated the impact of nesiritide on renal function in patients with ADHF, but no consistent conclusions can be drawn from them. A meta-analysis of five randomized studies with 1269 ADHF patients has demonstrated that intravenous administration of nesiritide significantly increase the risk of WRF and may cause renal toxicity [6]. However, in the subsequent clinical trials such as ASCEND-HF [4, 5], ROSE [7] and BNP-CARDS [8], it has been reported that low-dose (0.005 μg/kg/min without bolus) or standard-dose (2 μg/kg bolus followed by 0.01 μg/kg/min infusion) of nesiritide has no impact on renal function in patients with ADHF. In addition, a favorable renal effect has also been observed in some small studies [9, 10]. With these conflicting results, it is still difficult to determine the specific role of nesiritide in renal function of ADHF patients. To answer the question, we performed an updated meta-analysis of randomized controlled trials (RCT) to evaluate the renal effect of nesiritide in adult patients with ADHF, as compared with other anti-heart failure agents or placebo.

## Methods

### Literature Search and Study Selection

Pertinent articles were systematically searched (until November 30, 2014) in PubMed, Medline and Cochrane Library by two trained investigators. The following search strategy developed according to Yan et al [11] was utilized without any restrictions: (nesiritide) and (decompensated heart failure). Backward snowballing was also performed to search potentially relevant articles from the references of retrieved articles and pertinent reviews.

An article was eligible for inclusion if it met the following criteria: 1) original articles; 2) RCT design; 3) adult (age ≥ 18 years) participants with ADHF; 4) nesiritide administered through intravenous infusion; 5) reporting the incidence of WRF or mean change of serum creatinine (SCr) from baseline. Articles which did not meet the aforementioned criteria were excluded from analysis. This meta-analysis was performed according to the Preferred Reporting Items for Meta-Analyses (PRISMA) statement checklist.

### Data Extraction and Quality Assessment

Two authors independently extracted the pertinent data for the meta-analysis, with divergences resolved by discussion. The following information was abstracted from each study: characteristics of each study (authors, year of publication, journal, country, study design), characteristics of participants (number, sex, average age, ethnicity), intervention and control treatments (bolus dose, infusion dose and duration) and clinical outcomes (incidence of WRF and peak mean change of SCr from baseline during the treatment of nesiritide). If several articles reported the same clinical trial, the one with the most complete data was included in analysis. And if a trial was designed with three or more than three groups, the control group was considered as a whole in the overall analysis and single group was considered separately in subgroup analysis.

Two authors independently evaluated, with disagreement resolved by discussion, the risk of bias for the included RCTs in accordance with the following criteria developed by the Cochrane risk of bias tool: random sequence generation, allocation concealment, blinding of participants and personnel, blinding of outcome assessment, incomplete outcome data, selective reporting and other sources of bias.

### Statistical Analysis

All the statistical analyses were performed with the use of STATA 12.0 (Stata Corp, College Station, Texas). Heterogeneity was evaluated using chi-square test (*P* ≤ 0.10 indicating significant heterogeneity) and I^2^ test. The relationship between nesiritide and incidence of WRF was analyzed using Mantel-haenszel (M-H) fixed-effect model (FFE) to calculate the relative risk (RR) and 95% confidence interval (CI), when there was no significant heterogeneity among the included studies; otherwise, a random-effect model was chosen. With regard to the analysis of peak mean change of SCr from baseline, inverse variance (IV) fixed-effect model was used to calculate the weight mean difference (WMD) and 95% CI without significant heterogeneity among the included studies; otherwise, a random-effect model was chosen. In order to evaluate the influence of different doses of nesiritide on the incidence of WRF, subgroup stratification analyses were also conducted. Sensitivity analysis was performed to evaluate the stability of results by exclusion of each study one by one. And publication bias was assessed with the use of funnel plots and Egger’s test.

## Results

### Literature Search

**Fig 1:**
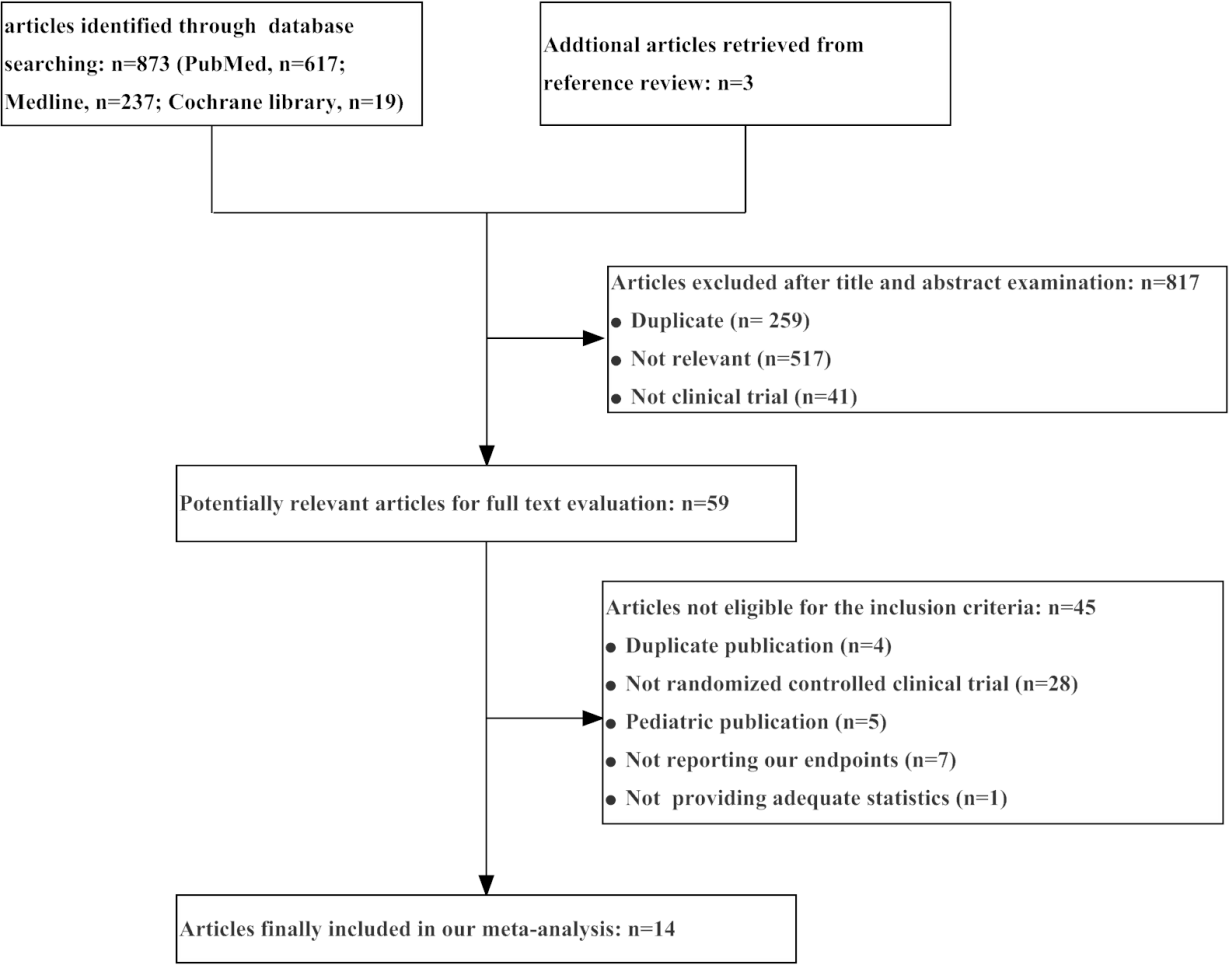
Flow diagram of study selection.

A total of 876 articles were yielded by database search and backward snowballing. Of the 876 articles, 817 were excluded after title and abstract examination, and 59 remained for full-text evaluation. Subsequently, 45 articles were excluded after full-text review, and 14 articles were eligible for final analysis. The process of literature search and reasons for exclusion were described in Fig 1.

### Study Characteristics

The main characteristics of the 15 included clinical trials in 14 articles are summarized in Table 1. All of the 15 clinical trials [5, 7, 8, 12–22] were RCT design published from 1999 to 2014 covering a total of 9623 patients with ADHF. Among the 15 RCTs, 13 reported the incidence of WRF including: four [7, 16, 18, 21] low-dose nesiritide RCTs (continuous infusion dose < 0.01 μg/kg/min), four [5, 8, 17, 22] standard-dose nesiritide RCTs (2 μg/kg bolus followed by 0.01 μg/kg/min infusion) and five [12–15] high-dose nesiritide RCTs (continuous infusion dose > 0.01 μg/kg/min). Renal function was considered to be worsened when SCr increased > 0.5mg/dL in eight studies, SCr increased > 0.3mg/dL in two studies, SCr increased ≥ 20% of baseline in one study, SCr increased ≥ 10% of baseline in one study, and glomerular filtration rate (GFR) decreased > 25% in one study. Peak mean change of SCr from baseline during the treatment of nesiritide was described in six [14, 15, 18–20, 22] studies of which most were used low-dose or standard-dose nesiritide.

**Table 1:** Characteristics of the included studies.

Author/Year	Design	Control	Blous dose (μg/kg)	Continuous infusion dose (μg/kg/min)	Length of infusion	Change of SCr mean±SD (μmol/L)	WRF definition	Total	WRF event (nesiritide)	WR event (control)
Mill, et al, (704.311), 1999, [12]	RCT	Placebo	0.25/0.50/1.00	0.015/0.06/0.03	24hrs	NA	Increase of SCr>0.5mg/dL	103	15/74	4/29
Colucci, et al (704.325), 2000, [13]	RCT	Placebo	0.30/0.60	0.015/0.03	6 hrs-5days	NA	Increase of SCr>0.5mg/dL	127	15/85	2/42
Colucci, et al (704.326), 2000, [13]	RCT	Standard care	0.30/0.60	0.015/0.03	2.3–283.2hrs/ 2.2–169.0hrs	NA	Increase of SCr>0.5mg/dL	305	36/203	9/102
VMAC, 2002, [14]	RCT	Nitroglycerin	2.00	0.01/0.03	24hrs	0.0±36.2	Increase of SCr>0.5mg/dL	489	74/273	45/216
Burger, et al, 2002, [15]	RCT	Dobutamine	No	0.015/0.03	24hrs-14days	8.8±35.4 8.8±53.0	Increase of SCr>0.5mg/dL	245	29/162	9/83
Yancy, et al (FUSION I) 2006, [16]	RCT	Standard care	1.00/2.00	0.005/0.01	12weeks	NA	Increase of SCr>0.5mg/dL	136	33/95	18/41
Witteles, et al, 2007, [8]	RCT	Placebo	2.00	0.01	48hrs	NA	Increase of SCr≥20% of baseline	75	9/39	9/36
Yancy, et al (FUSION II) 2008, [17]	RCT	Placebo	2.00	0.01	12weeks	NA	Increase of SCr>0.5mg/dL	911	194/ 605	119/306
Owan, et al, 2008, [22]	RCT	Standard care	2.00	0.01	48hrs	0.0±2.7	Increase of SCr>0.5mg/dL	55	2/28	3/27
Zhao, et al, 2010, [18]	RCT	Sodium nitroprusside	0.50	0.0075	72hrs	-6.6±26.7	Increase of SCr≥10% of baseline	55	0/27	1/28
Chow, et al, 2011, [19]	RCT	Nitroglycerin	2.00	0.01	48hrs	0.0±38.9	NA	NA	NA	NA
O'Connor, et al, 2011, [5]	RCT	Placebo	2.00	0.01	24hrs-7days	NA	Decrease of GFR>25%	6567	1032/ 3289	968/ 3278
Fu, et al, 2012, [21]	RCT	Standard care	No	0.0075–0.015	13days	NA	Increase of SCr>0.3mg/dL	140	16/70	14/70
Chen, et al, 2013, [7]	RCT	Placebo	No	0.005	72hrs	NA	Increase of SCr>0.3mg/dL	221	28/112	24/109
Pan, et al, 2014, [20]	RCT	Dobutamine	1.50	0.0075–0.01	24-72hrs	2.4±32.2/6.9±32.9	NA	105	NA	NA

Abbreviations: NA, Not available; GFR, Glomerular filtration rate; RCT, Randomized controlled trial; SCr, Serum creatinine SD, Standard deviation; WRF, Worsening renal function

### Quality Assessment

The risk of bias of included RCT studies is summarized in S1 Table. Seven items developed by Cochrane risk of bias tool were used to perform this evaluation. Most items for all included studies indicated low risk. However, five studies were open label design. On the whole, the RCT studies included in our meta-analysis had relatively high quality.

### Risk of Worsening Renal Function

**Fig 2:**
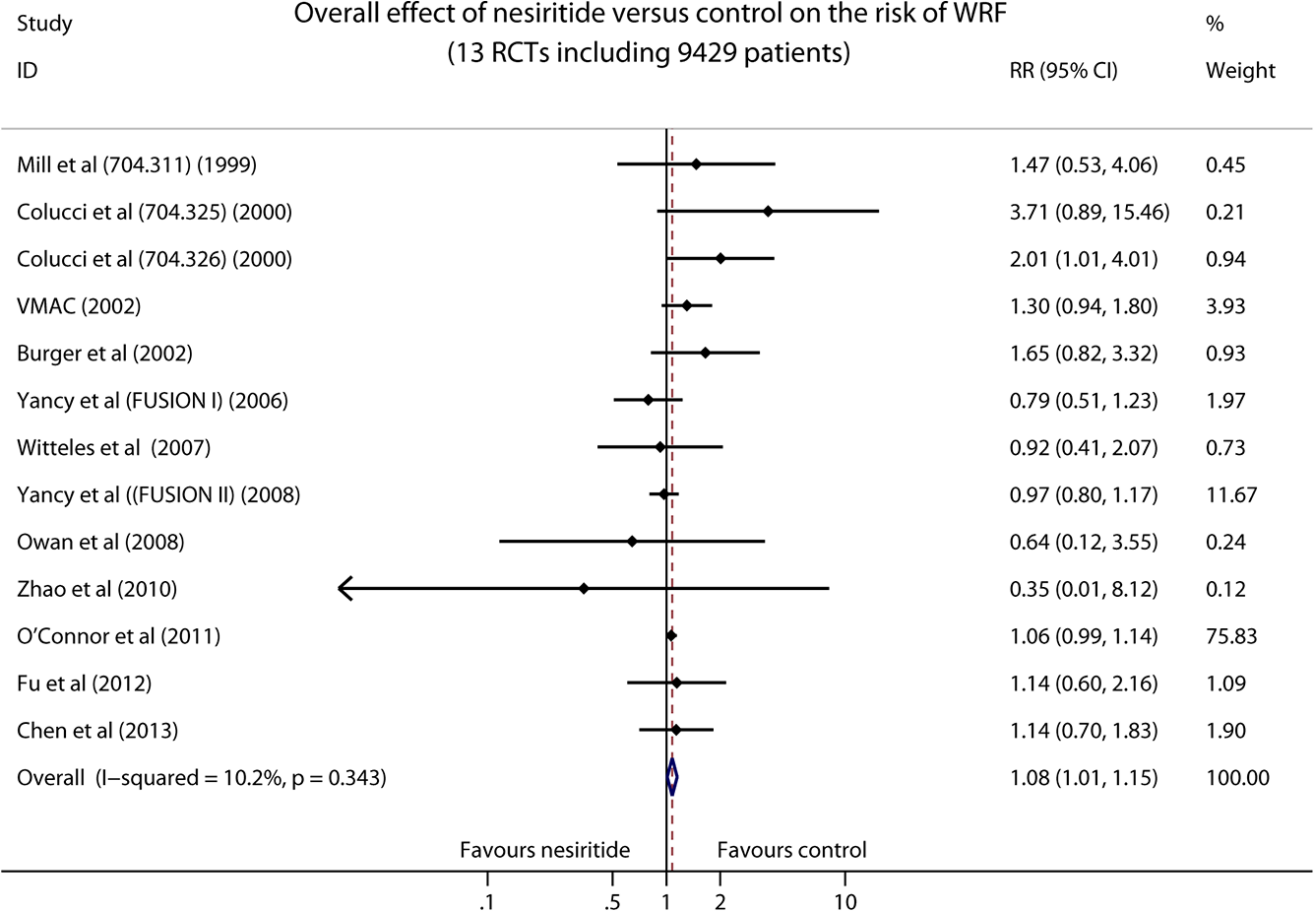
Forest plot depicting the overall effect of nesiritide on the risk of WRF in patients with ADHF.

A total of 13 nesiritide associated RCTs described the incidence of WRF in ADHF patients. As compared with non-nesiritide-based control therapies (dobutamine, nitroglycerin, sodium nitroprusside, standard care of ADHF and placebo), overall meta-analysis indicated that the use of nesiritide was associated with a significant increase in the risk of WRF (9429 patients, RR 1.08, 95% CI 1.01–1.15, *P* = 0.023; Fig 2). There was no significant heterogeneity among all the studies (I^2^ = 10.2%, *P* = 0.343). The funnel plot demonstrated a little asymmetry (Fig A in S1 File), but there was no significant publication bias detected by Egger’s test (*P* = 0.343; Fig B in S1 File).

**Fig 3:**
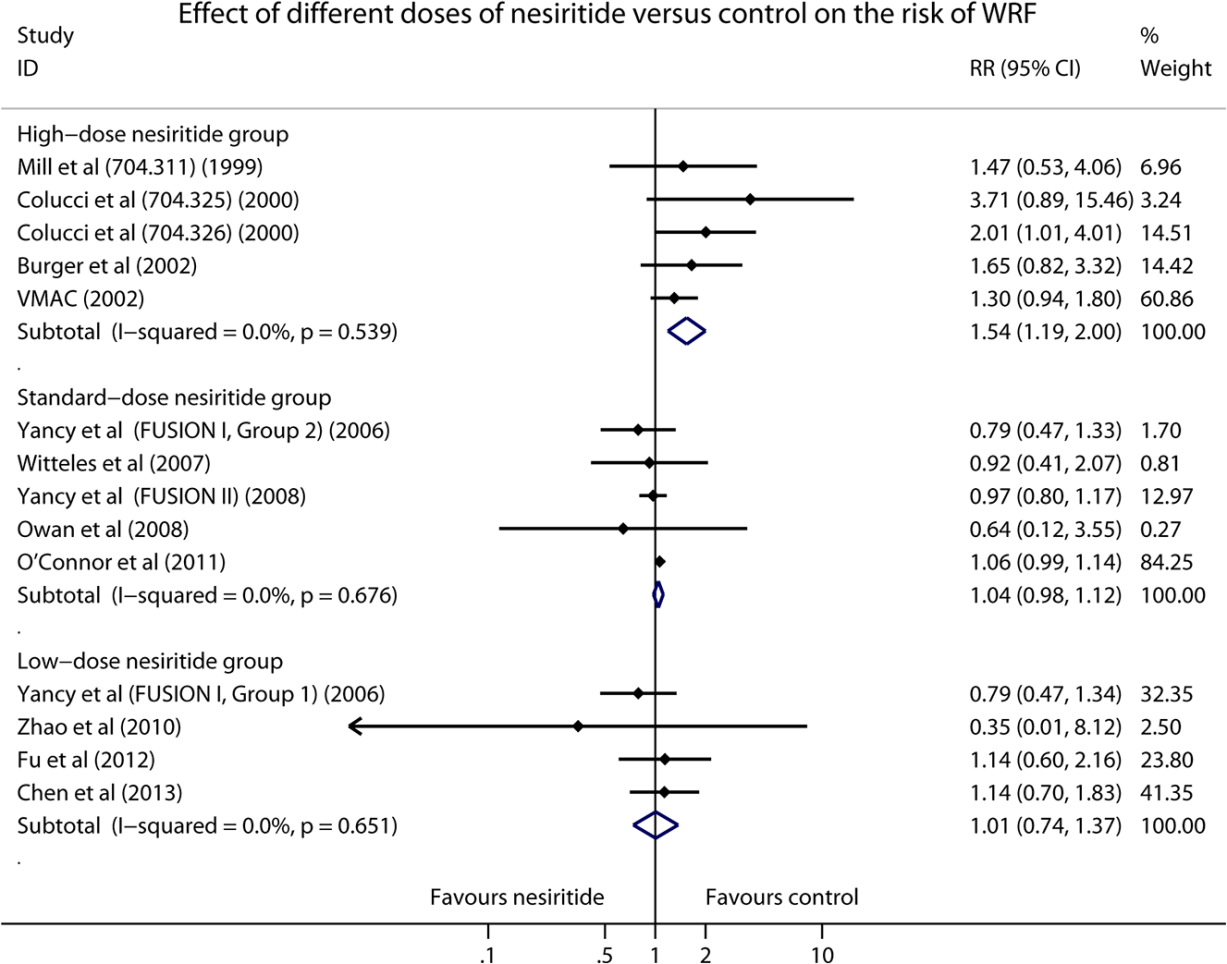
Forest plot depicting the effect of different doses of nesiritide on the risk of WRF in patients with ADHF.

To further investigate the relationship between continuous infusion doses of nesiritide and renal dysfunction, subgroup meta-analysis was performed. Five RCTs administered nesiritide in a high-dose manner. The data of analysis showed that high-dose nesiritide strongly increased the risk of renal dysfunction in patients with ADHF (1269 patients, RR 1.54, 95% CI 1.19–2.00, *P* = 0.001; Fig 3) without significant heterogeneity (I^2^ = 0.0%, *P* = 0.539). However, there was no significant influence of nesiritide on renal function in the standard-dose group including five RCTs or subgroups (7698 patients, RR 1.04, 95% CI 0.98–1.12, *P* = 0.213; Fig 3) without significant heterogeneity (I^2^ = 0.0%, *P* = 0.676). Furthermore, in the low-dose group consisted of four RCTs or subgroups, significantly renal benefit was not observed either (503 patients, RR 1.01, 95% CI 0.74–1.37, *P* = 0.968; Fig 3) and no significant heterogeneity was presented (I^2^ = 0.0%, *P* = 0.651).

**Fig 4:**
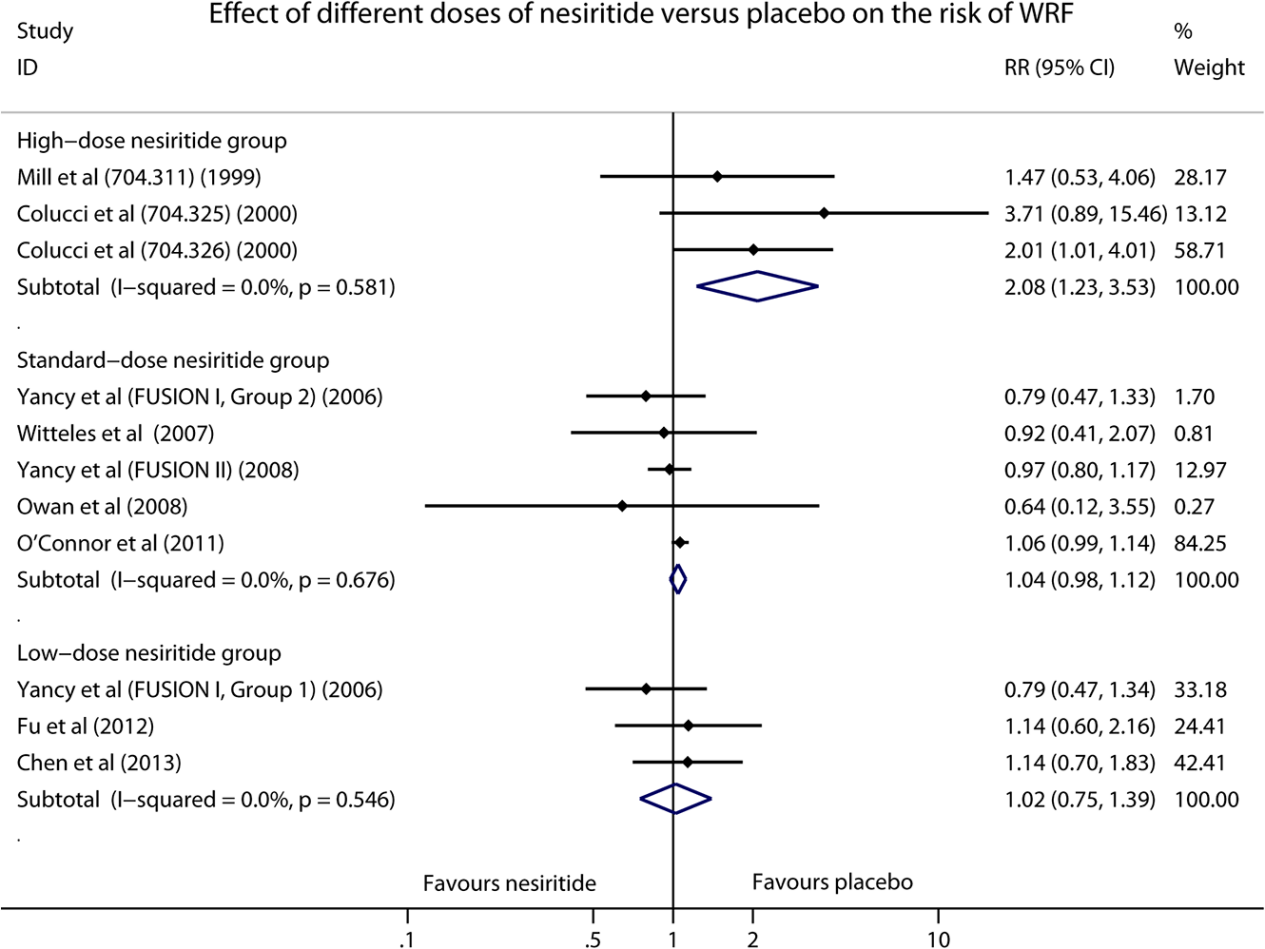
Forest plot depicting the effect of different doses of nesiritide on the risk of WRF in placebo (standard care) controlled trials.

**Fig 5:**
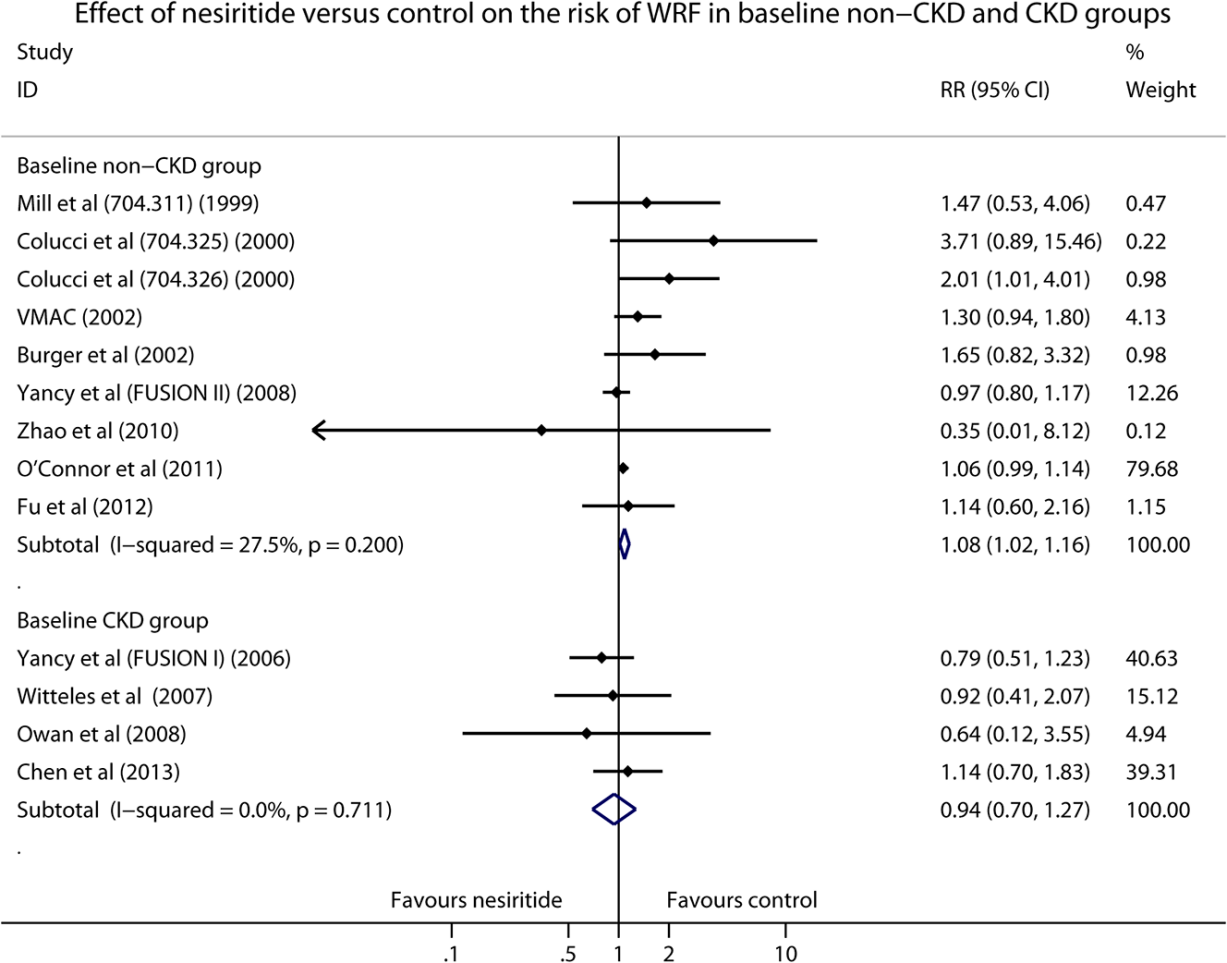
Forest plot depicting the effect of nesiritide on the risk of WRF in baseline non-CKD and CKD patients with ADHF.

We also conducted a subgroup analysis of placebo (standard care) controlled trials to partly exclude the potential drug-drug interactions by removing the active controlled studies which used dobutamine, nitroglycerin or sodium nitroprusside as control treatments. As shown in Fig 4, significant difference was found in high-dose nesiritide group (RR 2.08, 95% CI 1.23–3.53, *P* = 0.007; I^2^ = 0.0%, *P* = 0.581), but neither standard-dose (RR 1.04, 95% CI 0.98–1.12, *P* = 0.213; I^2^ = 0.0%, *P* = 0.676) nor low-dose nesiritide (RR 1.02, 95% CI 0.75–1.39, *P* = 0.884; I^2^ = 0.0%, *P* = 0.546) statistically increased the risk of WRF. Furthermore, to identify whether baseline chronic kidney disease (CKD) could influence the occurrence of WRF in patients with ADHF, a subgroup analysis based on the condition of baseline renal function was carried out. Interestingly, in comparison with control therapies, nesiritide significantly increased the risk of WRF in non-CKD patients (RR 1.08, 95% CI 1.02–1.16, *P* = 0.016; I^2^ = 27.5%, *P* = 0.200; Fig 5), but not in patients with CKD (RR 0.94, 95% CI 0.70–1.27, *P* = 0.680; I^2^ = 0.0%, *P* = 0.711; Fig 5).

### Mean Change of Serum Creatinine

**Fig 6:**
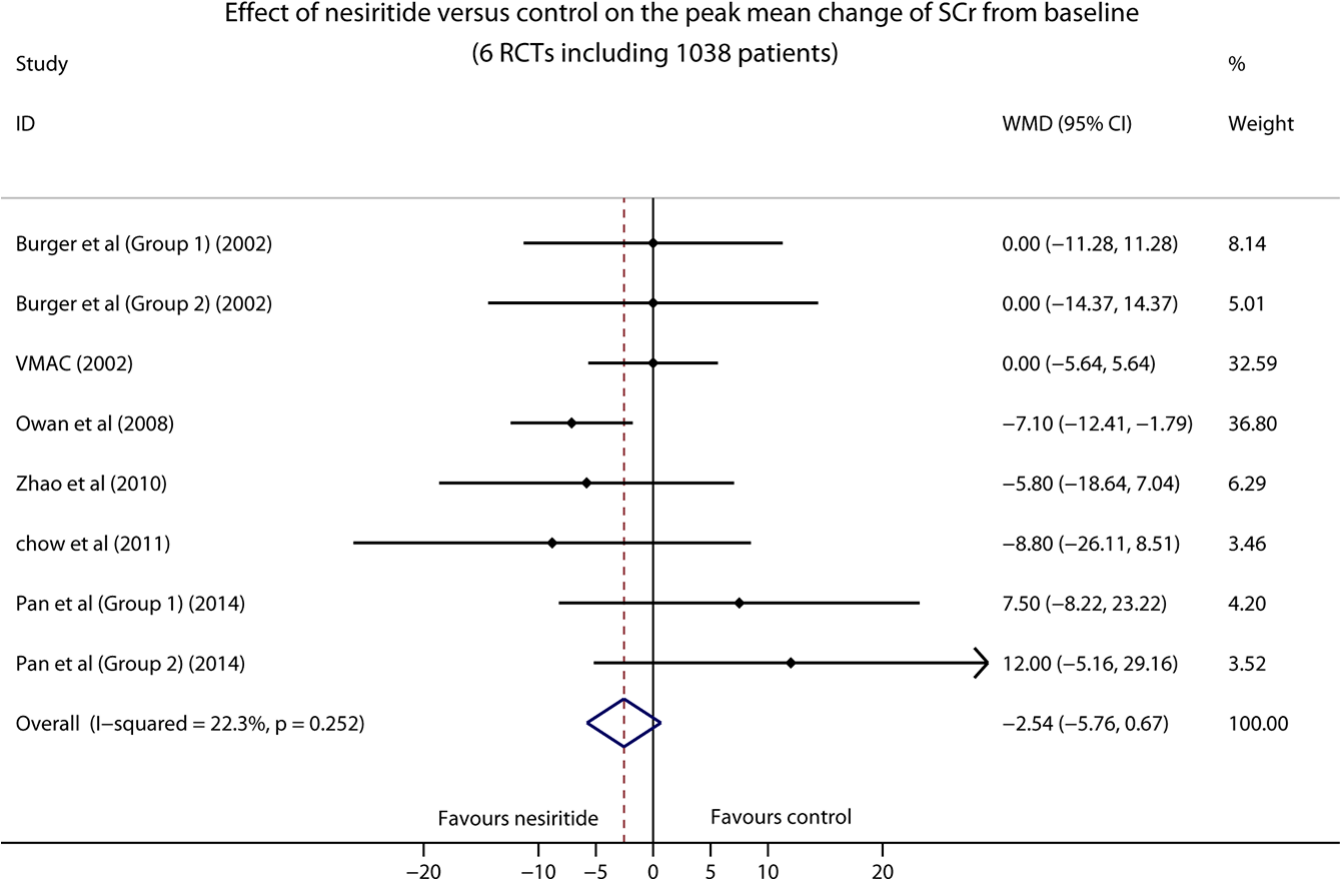
Forest plot depicting the effect of nesiritide on the peak mean change of SCr from baseline in patients with ADHF.

We collected the peak mean change of SCr from baseline during the intravenous infusion of nesiritide, to assess whether nesiritide had an impact on the concentration of SCr and renal function. A total of six RCTs involved in this endpoint and four of them administered nesiritide with standard-dose or low-dose. According to the result of meta-analysis, the concentration of SCr was not significantly different between nesiritide and control therapies (1038 patients, WMD -2.54, 95% CI -5.76–0.67, *P* = 0.121; Fig 6). Significant heterogeneity was not found among the studies (I^2^ = 22.3%, *P* = 0.252). Funnel plot did not show obvious asymmetry (Fig C in S1 File), and no publication bias was observed in Egger’s test (P = 0.278; Fig D in S1 File).

### Sensitivity Analysis

According to the results of sensitivity analysis, there was no substantial modification of our estimates after exclusion of individual study one by one.

## Discussion

Nesiritide, approved by the Food and Drug Administration in 2001, is a new therapy for ADHF with a potent effect on reducing pulmonary-capillary wedge pressure and systematic blood pressure [23]. However, there are conflicting data regarding the effect of nesiritide on renal function in patients with ADHF [5, 6]. Hence, this meta-analysis was designed to determine the specific role of nesiritide in renal function. The overall meta-analysis indicated that nesiritide may have a potential to impair renal function in patients with ADHF. Further subgroup analyses found that high-dose nesiritide strongly increased the risk of WRF, while this adverse effect was not observed in standard-dose and low-dose groups. In addition, the peak mean change of SCr from baseline was not found to be significantly different between nesiritide and control therapies.

On the whole, detrimental effect of nesiritide on renal function was found in our meta-analysis. On the basis of subsequent subgroup analyses, we speculate that the strongly association between high-dose nesiritide and WRF may play a critical role in the overall effect. A previous meta-analysis including five RCTs reported that the incidence of WRF was 50% more prevalent in the nesiritide group [6]. In accordance with our high-dose group, all the five RCTs in that meta-analysis used relatively high continuous infusion dose of nesiritide ranging from 0.015 to 0.06μg/kg/min [12–15]. Several possible reasons may explain this high-dose effect. First, systematic blood pressure may play an important role in renal function [24]. Previous studies have found that ADHF patients with hypotension may have higher risk of renal dysfunction than those without hypotension and the use of nesiritide could produce an inverse dose-dependent effect on blood pressure [4, 25, 26]. Hence, a higher dose of nesiritide probably produces a greater decrease of systemic blood pressure to reduce the renal perfusion and further enhance the occurrence of renal dysfunction. The other explanation is that greater reduction of blood pressure with high-dose nesiritide may excessively activate the counter-regulatory mechanisms such as sympathetic and renin-angiotensin systems to impair renal function [22, 27].

Our subgroup meta-analyses based on the administration dosages of nesiritide suggested that as compared with control therapies or placebo (standard care), standard-dose of nesiritide neither deteriorated nor improved the renal function of patients with ADHF. The conclusions of most RCTs included in these subset analyses were in accordance with our result [5, 8, 16, 17, 22]. Pathophysiologically, nesiritide not only could directly influence the glomerular arterioles, mesangial cells and renal tubules to improve renal function, but also could indirectly regulate the systemic hemodynamics, renin-angiotensin-aldosterone axis and sympathetic system to worsen renal function [28–31]. Therefore, the interaction of these two opposite effects may determine the final influence of nesiritide on renal function. The administration of nesiritide with standard-dose probably balances the direct and indirect effects. Consequently, no significantly renal effect was observed in the standard-dose group.

There are conflicting data about the effect of low-dose nesiritide on renal function. All the RCTs analyzed in our meta-analysis have not found that low-dose nesiritide had a potential to improve renal function in patients with ADHF [7, 16, 18, 21]. However in some non-randomized pilot studies, low dose nesiritide has been demonstrated to have a favorable effect on renal function in patients with acute heart failure or undergoing cardiac surgery [9, 10, 32]. Possible explanations for this discrepancy seem to be limited sample size as well as relatively low quality of study design in these small studies. It is well known that RCT is a study design with relatively high quality which is the best way to evaluate clinical trials and avoid many types of bias [33]. Four RCTs in our analysis avoided possible bias to a large extent without significant heterogeneity. Therefore, the conclusion in our meta-analysis is likely to be more accurate. However, it was worthy to note that there were only four clinical trials with 503 patients in our analysis. Hence, whether low-dose nesiritide could improve renal function in patients with ADHF needs to be further evaluated.

Data from our subgroup analysis indicated that nesiritide significantly increased the risk of WRF in non-CKD patients, however the adverse effect was not observed in ADHF patients with pre-existing CKD. This finding is a little confusing. But it was noteworthy that all the studies in CKD group administered nesiritide with either standard or low dose [7, 8, 16, 22]. On the contrary, a large proportion of RCTs [12–15] in non-CKD group used high-dose nesiritide for the management of patients with ADHF. Hence, the potential high-dose associated WRF might partly explain the interesting result. In the future, other RCTs may be required to further identify whether the renal effects of nesiritide therapy are different between CKD and non-CKD ADHF patients.

SCr is still the main biomarker for predicting the renal function of patients [34]. In our meta-analysis, the peak mean change of SCr from baseline was obtained from six RCTs and no statistical difference was found. This effect may be partly attributed to that most of the included studies administered nesiritide with standard-dose or low-dose. However, due to the limited number of clinical trials, the subgroup analysis was not performed to further investigate whether there is a dose-dependent effect of nesiritide on the concentration of SCr. Cystatin C is a novel and more sensitive marker of early renal insufficiency [35]. A few studies have demonstrated that cystatin C could provide more precise prediction of WRF than SCr in patients with heart failure [36–38]. Two RCTs in our meta-analysis as well as a retrospective study of ASCEND-HF, of which all administered nesiritide with standard dose, used cystatin C to evaluate the renal function of ADHF patients, and there was no significant difference between nesiritide and control therapies in all of them [7, 22, 39]. Therefore, these findings appear to reassure the renal safety of nesiritide with standard-dose or low-dose.

All the studies included in our meta-analysis were randomized design and had relatively high quality. Hence, our conclusions were relatively reliable, while there were several limitations in our study. Firstly, small sample size and limited number of clinical trials in subgroup analyses might produce bias. Secondly, the definition of WRF in included studies was not completely consistent, which might result in clinical and statistical heterogeneity. Thirdly, duration of intravenous nesiritide infusion was not same in each study, which might influence the clinical outcome. Fourthly, besides placebo (standard care), the control therapies also included dobutamine, nitroglycerin, and sodium nitroprusside. Therefore drug-drug interactions were not to be fully considered and might impact clinical outcome.

In conclusion, the present meta-analysis demonstrates that nesiritide may have a dose-dependent effect on renal function in patients with ADHF. High-dose nesiritide may increase the risk of WRF, but neither standard-dose nor low-dose nesiritide is likely to have a significant impact on renal function in patients with ADHF. These findings could be helpful to optimize the use of nesiritide in clinical practice. In the future, more powered RCTs will be needed to confirm these findings. Additionally, whether low-dose nesiritide could improve the renal function in patients with ADHF also requires to be addressed.

## Supporting Information

S1 PRISMA Checklist PRISMA checklist of this meta-analysis. (DOC)

S1 File Supporting information of figures. Fig A. Funnel plot of included studies for risk of WRF analysis. Fig B. Egger’s publication bias plot of included studies for risk of WRF analysis. Fig C. Funnel plot of included studies for peak mean change of SCr from baseline analysis. Fig D. Egger’s publication bias plot of included studies for peak mean change of SCr from baseline analysis.
(DOCX)

S1 Table Risk of bias assessment for included studies. (DOCX)

## References

[1] Butler J, Chirovsky D, Phatak H, McNeill A, Cody R. Renal Function, Health Outcomes, and Resource Utilization in Acute Heart Failure A Systematic Review. Circ Heart Fail. 2010;3(6):726–45. 10.1161/CIRCHEARTFAILURE.109.920298 21081740

[2] Metra M, Cotter G, Gheorghiade M, Dei Cas L, Voors AA. The role of the kidney in heart failure. Eur Heart J. 2012;33(17):2135–42. 10.1093/eurheartj/ehs205 22888113

[3] Nohria A, Hasselblad V, Stebbins A, Pauly DF, Fonarow GC, Shah M, et al Cardiorenal interactions: insights from the ESCAPE trial. J Am Coll Cardiol. 2008;51(13):1268–74. 10.1016/j.jacc.2007.08.072 18371557

[4] van Deursen VM, Hernandez AF, Stebbins A, Hasselblad V, Ezekowitz JA, Califf RM, et al Nesiritide, renal function, and associated outcomes during hospitalization for acute decompensated heart failure: results from the Acute Study of Clinical Effectiveness of Nesiritide and Decompensated Heart Failure (ASCEND-HF). Circulation. 2014;130(12):958–65. 10.1161/CIRCULATIONAHA.113.003046 25074507

[5] O'Connor C, Starling R, Hernandez A, Armstrong P, Dickstein K, Hasselblad V, et al Effect of nesiritide in patients with acute decompensated heart failure. New Engl J Med. 2011;365(1):32–43. 10.1056/NEJMoa1100171 21732835

[6] Sackner-Bernstein JD, Skopicki HA, Aaronson KD. Risk of worsening renal function with nesiritide in patients with acutely decompensated heart failure. Circulation. 2005;111(12):1487–91. 15781736 10.1161/01.CIR.0000159340.93220.E4

[7] Chen HH, Anstrom KJ, Givertz MM, Stevenson LW, Semigran MJ, Goldsmith SR, et al Low-dose dopamine or low-dose nesiritide in acute heart failure with renal dysfunction: the ROSE acute heart failure randomized trial. JAMA. 2013;310(23):2533–43. 10.1001/jama.2013.282190 24247300 PMC3934929

[8] Witteles RM, Kao D, Christopherson D, Matsuda K, Vagelos RH, Schreiber D, et al Impact of Nesiritide on Renal Function in Patients With Acute Decompensated Heart Failure and Pre-Existing Renal DysfunctionA Randomized, Double-Blind, Placebo-Controlled Clinical Trial. J Am Coll Cardiol. 2007;50(19):1835–40. 17980248 10.1016/j.jacc.2007.03.071

[9] Chen HH, Sundt TM, Cook DJ, Heublein DM, Burnett JC. Low Dose Nesiritide and the Preservation of Renal Function in Patients With Renal Dysfunction Undergoing Cardiopulmonary-Bypass Surgery A Double-Blind Placebo-Controlled Pilot Study. Circulation. 2007;116(11 suppl):I-134-I-8. 10.1161/CIRCULATIONAHA.106.697250 17846293

[10] Riter HG, Redfield MM, Burnett JC, Chen HH. Nonhypotensive low-dose nesiritide has differential renal effects compared with standard-dose nesiritide in patients with acute decompensated heart failure and renal dysfunction. J Am Coll Cardiol. 2006;47(11):2334–5. 16750705 10.1016/j.jacc.2006.03.013

[11] Yan B, Peng L, Zhao X, Chung H, Li L, Zeng L, et al Nesiritide fails to reduce the mortality of patients with acute decompensated heart failure: An updated systematic review and cumulative meta-analysis. Int J Cardiol. 2014;177(2):505–9. 10.1016/j.ijcard.2014.08.078 25183537

[12] Mills RM, LeJemtel TH, Horton DP, Liang C-s, Lang R, Silver MA, et al Sustained hemodynamic effects of an infusion of nesiritide (human b-type natriuretic peptide) in heart failureA randomized, double-blind, placebo-controlled clinical trial. J Am Coll Cardiol. 1999;34(1):155–62. 10400005 10.1016/s0735-1097(99)00184-9

[13] Colucci WS, Elkayam U, Horton DP, Abraham WT, Bourge RC, Johnson AD, et al Intravenous nesiritide, a natriuretic peptide, in the treatment of decompensated congestive heart failure. New Engl J Med. 2000;343(4):246–53. 10911006 10.1056/NEJM200007273430403

[14] Young JB, Abraham W, Stevenson L, Horton D, Elkayam U, Bourge R. Intravenous nesiritide vs nitroglycerin for treatment of decompensated congestive heart failure-A randomized controlled trial. Jama-Journal of the American Medical Association. 2002;287(12):1531–40. 10.1001/jama.287.12.1531 11911755

[15] Burger AJ, Horton DP, LeJemtel T, Ghali JK, Torre G, Dennish G, et al Effect of nesiritide (B-type natriuretic peptide) and dobutamine on ventricular arrhythmias in the treatment of patients with acutely decompensated congestive heart failure: the PRECEDENT study. Am Heart J. 2002;144(6):1102–8. 12486437 10.1067/mhj.2002.125620

[16] Yancy CW, Singh A. Potential Applications of Outpatient< i> Nesiritide Infusions in Patients With Advanced Heart Failure and Concomitant Renal Insufficiency (from the Follow-Up Serial Infusions of Nesiritide [FUSION I] Trial). The American journal of cardiology. 2006;98(2):226–9. 16828598 10.1016/j.amjcard.2006.01.081

[17] Yancy CW, Krum H, Massie BM, Silver MA, Stevenson LW, Cheng M, et al Safety and Efficacy of Outpatient Nesiritide in Patients With Advanced Heart Failure Results of the Second Follow-Up Serial Infusions of Nesiritide (FUSION II) Trial. Circ Heart Fail. 2008;1(1):9–16. 10.1161/CIRCHEARTFAILURE.108.767483 19808265

[18] Zhao Q, Wu T-G, Lin Y, Li B, Luo J-Y, Wang L-X. Low-dose nesiritide improves renal function in heart failure patients following acute myocardial infarction. Heart Vessels. 2010;25(2):97–103. 10.1007/s00380-009-1171-0 20339970

[19] Chow SL, O'Barr SA, Peng J, Chew E, Pak F, Quist R, et al Modulation of Novel Cardiorenal and Inflammatory Biomarkers by Intravenous Nitroglycerin and Nesiritide in Acute Decompensated Heart Failure An Exploratory Study. Circ Heart Fail. 2011;4(4):450–5. 10.1161/CIRCHEARTFAILURE.110.958066 21576282

[20] Pan H-Y, Zhu J-H, Gu Y, Yu X-H, Pan M, Niu H-Y. Comparative effects of recombinant human brain natriuretic peptide and dobutamine on acute decompensated heart failure patients with differsent blood BNP levels. BMC Cardiovasc Disord. 2014;14(1):31. 24593826 10.1186/1471-2261-14-31 PMC3975880

[21] Fu S, Yi S, Zhu B, Wang L, Wang H, Bai Y, et al Efficacy and safety of a modified dosage regimen of nesiritide in patients older than 75 years with acute heart failure. Aging Clin Exp Res. 2012;24(5):524–9. 10.3275/8295 22395196

[22] Owan TE, Chen HH, Frantz RP, Karon BL, Miller WL, Rodeheffer RJ, et al The effects of nesiritide on renal function and diuretic responsiveness in acutely decompensated heart failure patients with renal dysfunction. J Card Fail. 2008;14(4):267–75. 10.1016/j.cardfail.2007.12.002 18474338 PMC2424167

[23] Peacock WF, Emerman CL, Silver MA, Group PS. Nesiritide added to standard care favorably reduces systolic blood pressure compared with standard care alone in patients with acute decompensated heart failure. The American journal of emergency medicine. 2005;23(3):327–31. 15915407 10.1016/j.ajem.2004.11.002

[24] Voors AA, Davison BA, Felker GM, Ponikowski P, Unemori E, Cotter G, et al Early drop in systolic blood pressure and worsening renal function in acute heart failure: renal results of Pre‐RELAX‐AHF. Eur J Heart Fail. 2011;13(9):961–7. 10.1093/eurjhf/hfr060 21622980

[25] Gassanov N, Biesenbach E, Caglayan E, Nia A, Fuhr U, Er F. Natriuretic peptides in therapy for decompensated heart failure. Eur J Clin Pharmacol. 2012;68(3):223–30. 10.1007/s00228-011-1117-1 21901345

[26] Colucci WS. Nesiritide for the treatment of decompensated heart failure. J Card Fail. 2001;7(1):92–100. 11264555 10.1054/jcaf.2001.22999

[27] Gnanaraj JF, von Haehling S, Anker SD, Raj DS, Radhakrishnan J. The relevance of congestion in the cardio-renal syndrome. Kidney Int. 2013;83(3):384–91. 10.1038/ki.2012.406 23254894

[28] Epstein FH, Levin ER, Gardner DG, Samson WK. Natriuretic peptides. New Engl J Med. 1998;339(5):321–8. 9682046 10.1056/NEJM199807303390507

[29] Houben AJ, Zander K, Leeuw PW. Vascular and renal actions of brain natriuretic peptide in man: physiology and pharmacology. Fundam Clin Pharmacol. 2005;19(4):411–9. 16011727 10.1111/j.1472-8206.2005.00336.x

[30] Brunner-La Rocca HP, Kaye DM, Woods RL, Hastings J, Esler MD. Effects of intravenous brain natriuretic peptide on regional sympathetic activity in patients with chronic heart failure as compared with healthy control subjects. J Am Coll Cardiol. 2001;37(5):1221–7. 11300426 10.1016/s0735-1097(01)01172-x

[31] Munagala VK, Burnett JC, Redfield MM. The natriuretic peptides in cardiovascular medicine. Curr Probl Cardiol. 2004;29(12):707–69. 15550914 10.1016/j.cpcardiol.2004.07.002

[32] Chen HH, AbouEzzeddine OF, Anstrom KJ, Givertz MM, Bart BA, Felker GM, et al Targeting the kidney in acute heart failure: can old drugs provide new benefit? Renal Optimization Strategies Evaluation in Acute Heart Failure (ROSE AHF) Trial. Circ Heart Fail. 2013;6(5):1087–94. 10.1161/CIRCHEARTFAILURE.113.000347 24046475 PMC3878083

[33] Pogue J, Yusuf S. Overcoming the limitations of current meta-analysis of randomised controlled trials. The Lancet. 1998;351(9095):47–52. 10.1016/S0140-6736(97)08461-4 9433436

[34] Legrand M, Jacquemod A, Gayat E, Collet C, Giraudeaux V, Launay J-M, et al Failure of renal biomarkers to predict worsening renal function in high-risk patients presenting with oliguria. Intensive Care Med. 2015;41(1):68–76. 10.1007/s00134-014-3566-3 25465906

[35] Lassus J, Harjola V-P. Cystatin C: a step forward in assessing kidney function and cardiovascular risk. Heart Fail Rev. 2012;17(2):251–61. 10.1007/s10741-011-9242-6 21431356

[36] McAlister FA, Ezekowitz J, Tarantini L, Squire I, Komajda M, Bayes-Genis A, et al Renal dysfunction in patients with heart failure with preserved versus reduced ejection fraction impact of the new chronic kidney disease-epidemiology collaboration group formula. Circ Heart Fail. 2012;5(3):309–14. 10.1161/CIRCHEARTFAILURE.111.966242 22441773

[37] Inker LA, Schmid CH, Tighiouart H, Eckfeldt JH, Feldman HI, Greene T, et al Estimating glomerular filtration rate from serum creatinine and cystatin C. New Engl J Med. 2012;367(1):20–9. 10.1056/NEJMoa1114248 22762315 PMC4398023

[38] Inker LA, Shaffi K, Levey AS. Estimating Glomerular Filtration Rate Using the Chronic Kidney Disease-Epidemiology Collaboration Creatinine Equation Better Risk Predictions. Circ Heart Fail. 2012;5(3):303–6. 10.1161/CIRCHEARTFAILURE.112.968545 22589364 PMC3386522

[39] Tang WW, Dupont M, Hernandez AF, Voors AA, Hsu AP, Felker GM, et al Comparative Assessment of Short-Term Adverse Events in Acute Heart Failure With Cystatin C and Other Estimates of Renal Function: Results From the ASCEND-HF Trial. JACC: Heart Failure. 2015;3(1):40–9. 10.1016/j.jchf.2014.06.014 25453534

