# Supplementary material for: The Dose-Dependent Effect of Nesiritide on Renal Function in Patients with Acute Decompensated Heart Failure: A Systematic Review and Meta-Analysis of Randomized Controlled Trials
Source: PLoS One. 2015 Jun 24;10(6):e0131326. doi: 10.1371/journal.pone.0131326 (PMC4479574; doi:10.1371/journal.pone.0131326)
Supplement: S1 File — Fig A. Funnel plot of included studies for risk of WRF analysis. Fig B. Egger’s publication bias plot of included studies for risk of WRF analysis. Fig C. Funnel plot of included studies for peak mean change of SCr from baseline analysis. Fig D. Egger’s publication bias plot of included studies for peak mean change of SCr from baseline analysis. (DOCX) [file pone.0131326.s002.docx]

**Fig. A. Funnel plot of included studies for risk of WRF analysis.
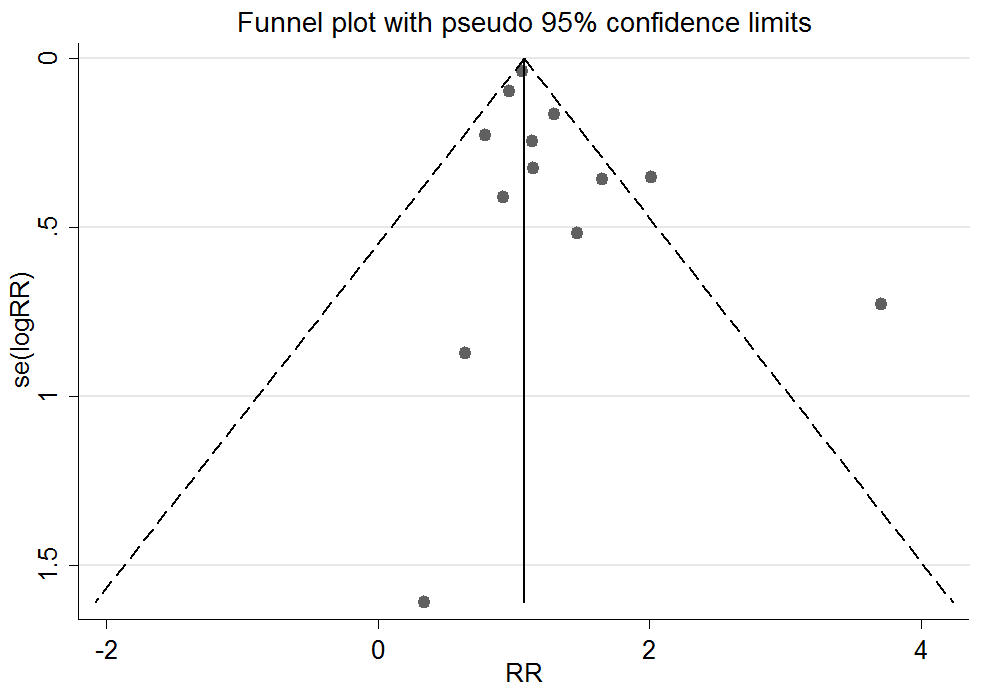
**

**Fig. B. Egger’s publication bias plot of included studies for risk of WRF analysis.**

**
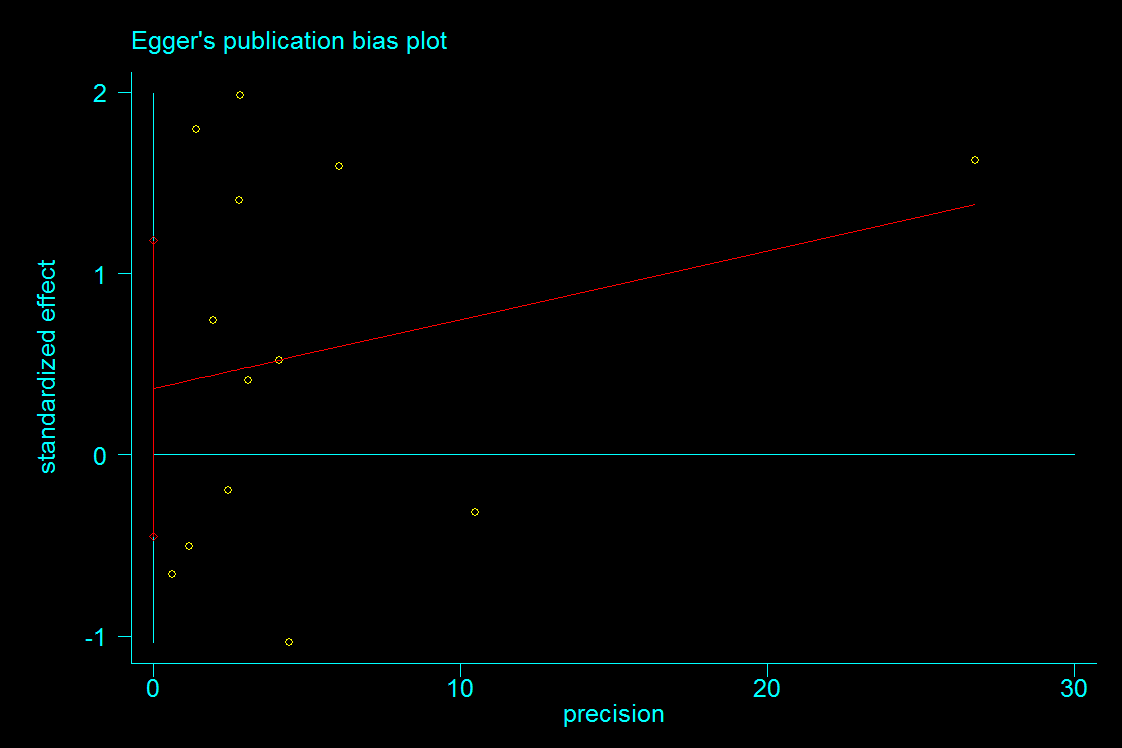
**

**Fig. C. Funnel plot of included studies for peak mean change of SCr from baseline analysis.**

**
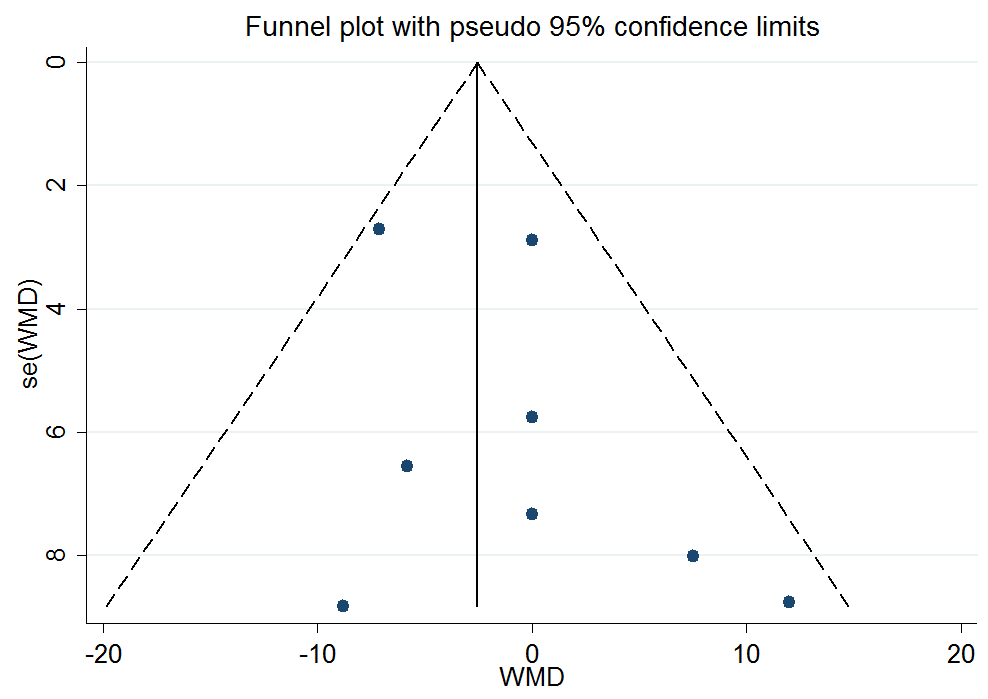
**

**Fig. D. Egger’s publication bias plot of included studies for peak mean change of SCr from baseline analysis.**

**
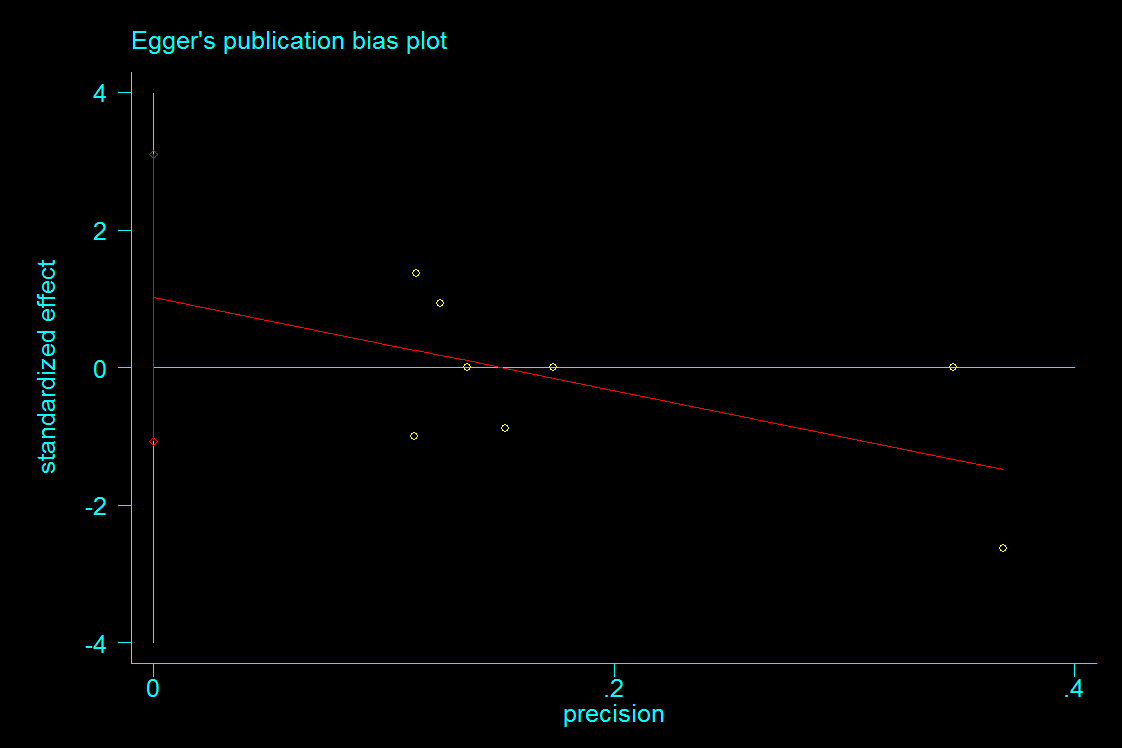
**
