# Supplementary material for: The Dose-Dependent Effect of Nesiritide on Renal Function in Patients with Acute Decompensated Heart Failure: A Systematic Review and Meta-Analysis of Randomized Controlled Trials
Source: PLoS One. 2015 Jun 24;10(6):e0131326. doi: 10.1371/journal.pone.0131326 (PMC4479574; doi:10.1371/journal.pone.0131326)
Supplement: S1 Table — (DOCX) [file pone.0131326.s003.docx]

**S1 Table. Risk of bias assessment for included studies**

| **Author** | **Random sequence generation** | **Allocation concealment** | **Blinding of participants and personnel** | **Blinding of outcome** | **Incomplete outcome data** | **Selective reporting** | **Other sources of bias** | **Overall risk of bias** |
| --- | --- | --- | --- | --- | --- | --- | --- | --- |
| Mill, et al | unclear | unclear | unclear | low | low | unclear | low | unclear |
| Colucci , et al (704.325) | Low | Low | Low | Low | Low | Low | Low | Low |
| Colucci , et al (704.326) | Low | Low | High | Low | Low | Low | Low | High |
| VMAC | Low | Low | Low | Low | Low | Low | Low | Low |
| Burger , et al | Unclear | Unclear | High | Low | Low | Unclear | Low | High |
| Yancy (FUSION I), et al | Unclear | Unclear | High | Low | Low | Unclear | Low | High |
| Witteles , et al | Low | Unclear | Low | Low | Low | Low | Low | Unclear |
| Yancy (FUSION II), et al | Low | Low | Low | Low | Low | Low | Low | Low |
| Owan , et al | Uclear | Unclear | Low | Low | Unclear | Low | Low | Unclear |
| Zhao , et al | Low | Unclear | Unclear | Low | Low | Low | Low | Unclear |
| Chow , et al | Unclear | Unclear | Low | Low | Low | Llow | Low | Unclear |
| O'Connor , et al | Low | Unclear | Low | Unclear | Low | Low | Low | Unclear |
| Fu , et al | Unclear | Unclear | High | Low | Low | Low | Low | High |
| Chen , et al | Low | Unclear | Low | Low | Low | Low | Low | Unclear |
| Pan, et al | Low | Unclear | High | Low | Low | Low | Low | High |
